# Determination of the genome-scale metabolic network of *Bartonella quintana* str. Toulouse to optimize growth for its use as chassis for synthetic biology

**DOI:** 10.3389/fbioe.2025.1527084

**Published:** 2025-03-27

**Authors:** Emilio Garrote-Sánchez, Andrés Moya, Rosario Gil

**Affiliations:** ^1^ Evolutionary Genetics, Institute for Integrative Systems Biology (I2SysBio), University of Valencia and Spanish Research Council, Valencia, Spain; ^2^ Genomic and Health Area, Foundation for the Promotion of Sanitary and Biomedical Research of the Valencia Region, Valencia, Spain

**Keywords:** *Bartonella quintana*, genome-scale metabolic models (GEMSs), proteomics, endosymbiont, Selective media

## Abstract

**Introduction:**

Genetically enhanced microorganisms have wide applications in different fields and the increasing availability of omics data has enabled the development of genome-scale metabolic models (GEMs), which are essential tools in synthetic biology. Bartonella quintana str. Toulouse, a facultative intracellular parasite, presents a small genome and the ability to grow in axenic culture, making it a potential candidate for genome reduction and synthetic biology applications. This study aims to reconstruct and analyze the metabolic network of B. quintana to optimize its growth conditions for laboratory use.

**Methods:**

A metabolic reconstruction of B. quintana was performed using genome annotation tools (RAST and ModelSEED), followed by refinement using multiple databases (KEGG, BioCyc, BRENDA). Flux Balance Analysis (FBA) was conducted to optimize biomass production, and in-silico knockouts were performed to evaluate growth yield under different media conditions. Additionally, experimental validation was carried out by testing modified culture media and performing proteomic analyses to identify metabolic adaptations.

**Results:**

FBA simulations identified key metabolic requirements, including 2-oxoglutarate as a crucial compound for optimal growth. In-silico knockouts of transport genes revealed their essentiality in nutrient uptake. Experimental validation confirmed the role of 2-oxoglutarate and other nutrients in improving bacterial growth, though unexpected decreases in viability were observed under certain supplemented conditions. Proteomic analysis highlighted differential expression of proteins associated with cell wall integrity and metabolic regulation

**Discussion:**

This study represents a step toward developing *B. quintana* as a viable chassis for synthetic biology applications. The reconstructed metabolic model provides a comprehensive understanding of *B. quintana*’s metabolic capabilities, identifying essential pathways and growth limitations. While metabolic predictions align with experimental results in key aspects, further refinements are needed to enhance model accuracy and optimize growth conditions.

## 1 Introduction

Genetically enhanced microorganisms have been used for decades in industry (Steensels and Verstrepen, 2014; Hemansi et al., 2022; Roy et al., 2023), agriculture (Icoz and Stotzky, 2008; Bharathi et al., 2011; Bommireddy et al., 2011; Tang and Zhang, 2023), and even in personalized medicine (Chua et al., 2017; Hwang et al., 2017). Its great potential is based on the development of a large number of bioinformatics applications that have facilitated the acquisition, storage, and analysis of extensive information on previously unknown microorganisms with considerably less effort. The increasing availability of massive data from -omics techniques, such as genomics, transcriptomics, or proteomics, enables the integration of all these elements into a unified mathematical model known as a Genome-Scale Metabolic Model (GEM). All these techniques are of great interest in synthetic biology, a discipline aimed at designing improved biological systems, where every process involved in growth and development can be controlled (Cheng and Lu, 2012; Smanski et al., 2016) producing one or more products of interest, whether biological or economical. In synthetic biology, the design cycle begins with conceptualizing the organism, followed by modeling and simulation to assess outcomes. Then, it proceeds with the development of a biological model consistent with the mathematical model, incorporating the genetic modifications necessary for its operation. Measurements in the modified organism help to refine the model; discrepancies with simulations may indicate design flaws that require modifications, while issues during the model construction may prompt a new testing cycle.

Two main approaches are commonly used to conduct synthetic biology experiments (Forster and Church, 2006; O’Malley et al., 2008): the top-down approach simplifies a complex model to achieve desired outcomes in a controlled environment, while the bottom-up approach meticulously constructs a refined model from basic components. Top-down experiments often start from well-studied model organisms such as *Escherichia coli* (Csörgo et al., 2012; Kurokawa and Ying, 2019) or *Bacillus subtilis* (Suárez et al., 2019). However, interactions due to the complexity of their genomes lead to significant and unexpected outcomes, such as an apparent gene redundancy, which may cause decreased performance when one of the genes is eliminated, or connections between genomic domains that worsen functionality after the removal of a DNA region (Moya et al., 2009). A common approach to mitigate these issues is to start with the simplest possible model to minimize the number of necessary changes, i.e., using organisms with a naturally small genome, close to what could be considered a minimal genome (Gil et al., 2004). Many such studies focus on the genomes of endosymbiotic organisms, as the presence of a host capable of supplying essential growth compounds results in a significant reduction of their genomes while preserving essential elements (Mehta et al., 2018; Madsen et al., 2022). However, it is known that during the reductive process, these symbiotic organisms lose their ability to grow in axenic cultures outside eukaryotic cells, which makes it more difficult to perform experimental studies.

Genus *Bartonella* consists of a large group within the class Alphaproteobacteria that maintain different symbiotic relationships with their hosts (Minnick and Anderson, 2014). Most of these species are facultative intracellular parasites, yet they can be grown *in vitro*, which makes them good candidates for use in laboratory experiments. Consistent with their host-associated lifestyle, all identified *Bartonella* species have small genomes, between 1.4 and 2.6 Mb, and have a region of high plasticity (Saenz et al., 2007; Engel et al., 2011), in which they present xenologous genes involved in their interaction with the host. These bacteria are transmitted via hematophagous insects and can establish chronic infections in several mammalian species, invading endothelial cells and erythrocytes. Some species can infect humans, although they are considered opportunistic pathogens.

Genetic modification studies have been carried out on these organisms, so editing their genomes is possible (Battisti and Minnick, 1999; Riess et al., 2004; MacKichan et al., 2008; Rolain et al., 2013). Since their infection mechanism has also been characterized (Saenz et al., 2007), it would be possible to modify their host specificity and infective capacity. Based on this, it has been proposed that *Bartonella* is a viable candidate species for the reduction of its genome in order to design a simplified genome without pathogenic capacity to which genes involved in some function of interest can be incorporated, with the aim of expressing them in mammalian cells in a stable but transient way (Klasson and Andersson, 2010). For this purpose, we chose *Bartonella quintana* str. Toulouse because it possesses one of the smallest genomes among the genus (Alsmark et al., 2004), and its genome has been successfully modified (MacKichan et al., 2008). Although *Bartonella bacilliformis*, with a smaller genome (Guillen et al., 2016), was also successfully modified (Battisti and Minnick, 1999), this work was performed on a non-sequenced mutant strain, and no further modifications have been reported. *B. quintana* is a fastidious organism that needs 12–14 days to obtain visible colonies when growing on chocolate agar plates in a 5% CO_2_ atmosphere. That makes it challenging to obtain enough cells to perform experiments, which prompted us to develop a metabolic model to try to improve its performance in laboratory culture.

## 2 Materials and methods

### 2.1 Reconstruction of the genome-scale metabolic model (GEM) of *Bartonella quintana*

We retrieved from the RefSeq database at NCBI the genomes of *B. quintana* str Toulouse (accession number NC_005955.1) and *Bartonella henselae* str. BM1374163 (accession number NZ_HG965802.1), the representative strain of this species. *B. henselae* is the most closely related species to *B. quintana* within the genus (Alsmark et al., 2004). We used OrthoVenn3 (Sun et al., 2023) to visualize and compare both genomes to solve some annotation problems.

The metabolic network reconstruction and refinement of the model were performed in several steps. An initial annotation of the genome sequence with RAST (Rapid Annotation using Subsystem Technology; Aziz et al., 2008; Brettin et al., 2015) was made and provided to ModelSEED (Overbeek et al., 2014; Seaver et al., 2021) to obtain a draft metabolic network with a general biomass reaction that needs to be curated. Many databases were used to ensure accuracy in the gap-filling of this preliminary metabolic network: KEGG (Ogata et al., 1999; Kanehisa et al., 2016; Kanehisa et al., 2017), MetaCyc and BioCyc (Caspi et al., 2018) have extensive information on genes, reactions, and pathways; BLAST (Altschul et al., 1990; Altschul et al., 1997) allows to search for orthologs and pseudogenes; BRENDA (Jeske et al., 2019) and ExPASy (Gasteiger et al., 2003) enable the search for enzymatic activities absent in our model organism. The addition or removal of reactions and genes in the model was based on genomic information, taking into account all complete annotated protein-coding genes for which there was evidence of a given activity in this organism or another one of the same genus. Enzymes with a broad substrate spectrum capable of compensating for the absence of other proteins were also considered. All reactions that are part of unconnected modules were removed from the model to avoid inconsistencies.

Modifications were made to the draft metabolic model using the COBRApy module in Python (Ebrahim et al., 2013). We used MEMOTE to determine the quality of the obtained GEM (Lieven et al., 2020). Cytoscape (Shannon et al., 2003) was used to interactively visualize the model, perform various topological analyses of the network, and create high-quality illustrations to display the results.

### 2.2 Flux balance analysis (FBA)

The biomass composition is a crucial factor in genome-scale metabolic models, significantly influencing their predictive accuracy and effectiveness. Flux balance analysis was performed to determine the optimal metabolic flux distributions, maximizing the biomass reaction as an objective function under steady-state assumption (Orth et al., 2010). Since there is no biomass composition information available for the genus *Bartonella*, we compared the biomass reaction prediction for the model with the well-documented biomass composition of *E. coli* model iJO1366 as a reference to adjust the biomass composition of our model. The components present in iJO1366 and not in the *Bartonella* biomass reaction were added with the corresponding stoichiometry; after manual curation of the network, compounds that could not be produced by the model were removed from the biomass equation. The stoichiometric values for dNTPs were calculated using the BOFdat software (Lachance et al., 2019). Due to the lack of kinetic data, we performed two different analyses: one without any constraint to the model input and output, and another with the only restriction of an oxygen intake flux of 15 mmol/gDW/h^-1^, which is the maximum flux of oxygen established for *E. coli* (Varma and Palsson, 1994).

In addition, we performed a genomic analysis of the transporter components involved in the model. We also generated single and double *in silico* knockouts of genes associated with transport to determine the changes in growth yield. Both FBA and *in silico* mutation simulations were performed using COBRApy.

### 2.3 Microbiological techniques

*B. quintana* str Toulouse (CIP 1033739) was provided by the Biological Resource Center of Institut Pasteur (CRBIP). All experiments started by streaking *B. quintana* from 100 μL of one- or two-passage frozen stock onto chocolate agar plates, made with Columbia blood agar base (Oxoid) supplemented with 5% defibrillated ovine blood (ThermoFischer Scientific). 2-oxoglutarate (500 mg/L, K), thiamine (1 mg/L, T) and folate (0.01 mg/L, F) were added when indicated. All cultures were plated in triplicate and incubated at 35°C in a 5% CO_2_ chamber, and the cells were harvested after 7 days with brain-heart infusion (BHI) broth.

To determine the cell growth in the different media, we prepared plate cultures in triplicate, harvested all cells in each plate, and determined the optical density at 600 nm (OD_600_) to estimate the initial concentration. Subsequently, we prepared a series of dilutions in liquid BHI, ranging from 10^–1^ to 10^–7^, and duplicate control chocolate agar plates were inoculated with dilutions from 10^–3^ to 10^–7^ for the cell count of the initial culture.

**FIGURE 1:**
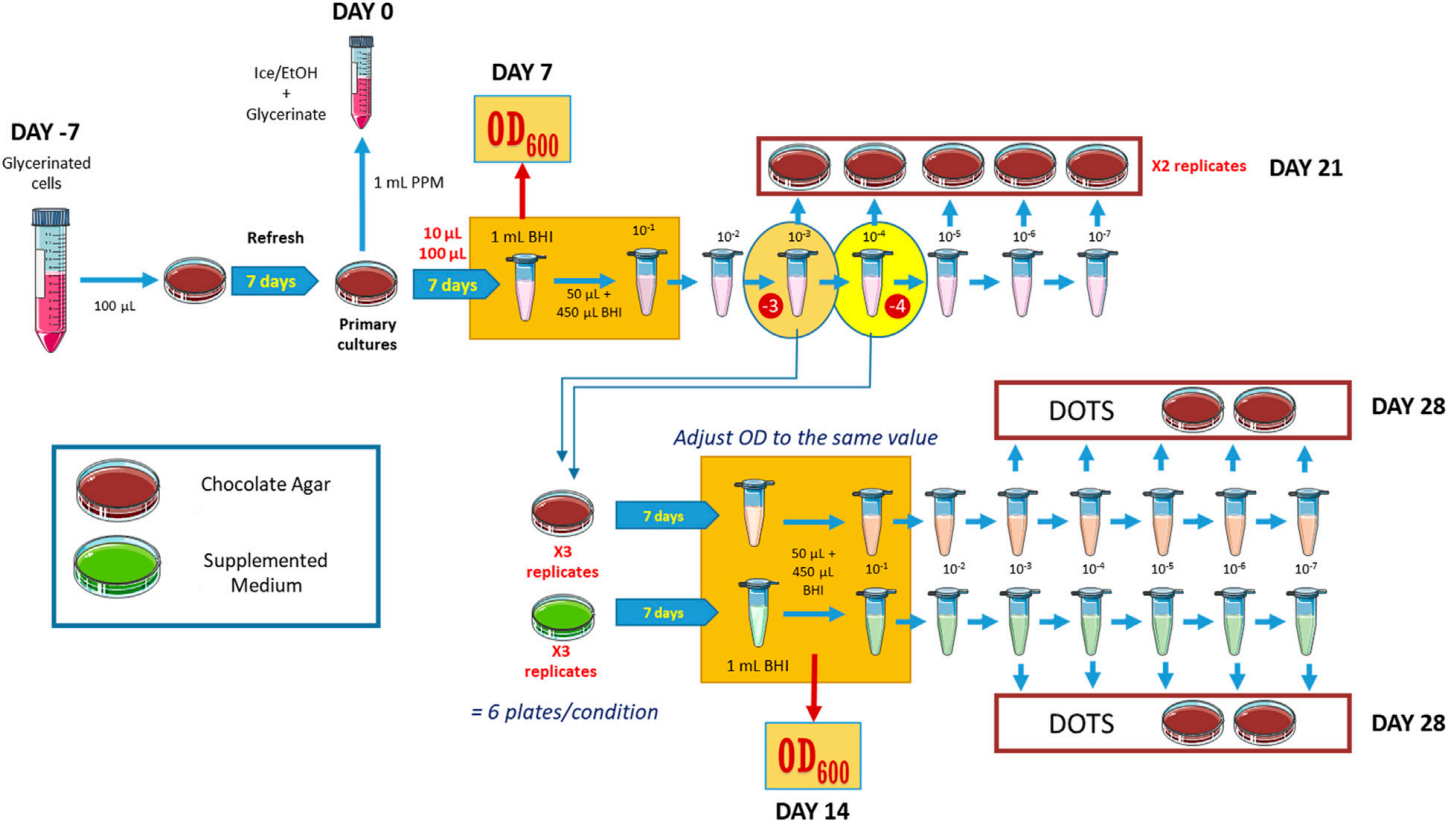
Experimental design to measure cell growth of *B. quintana* in selected media. OD measurements were made at the steps highlighted in orange. Details are given in the main text.

From the 10^–3^ and 10^–4^ dilutions obtained from these plates, triplicate inoculations of 100 μL were made for each condition under investigation, along with the control medium. After a 7-day incubation period, colonies were collected in liquid BHI, and the OD_600_ was determined. The optical densities of all conditions were normalized, and 5 μL dots of dilutions ranging from 10^–3^ to 10^–7^ from each culture were spotted in a gridded distribution to assess the proportion of the cells that remain viable. The protocol is schematized in Figure 1.

### 2.4 Total protein extraction for proteomic analysis

Cells grown in two agar plates were harvested in 2 mL of PBS buffer and centrifuged at 4°C for 10 min at 3500 g. The pellet was resuspended in 500 μL of PBS and washed three times under the same conditions. The sample was resuspended in 300 μL of solubilization buffer (8 M urea, 2 M thiourea, 4% V/V CHAPS, 20 M Tris, Roche Complete Protease Inhibitor 1x) to release and solubilize the total cellular proteins. Finally, the sample was centrifuged at 4°C for 5 min at 10 000 g, and the supernatant was collected.

A comparative proteomic analysis of samples obtained in the different culture conditions was conducted using the SWATH mass spectrometry method (Collins et al., 2017; Ludwig et al., 2018). Mass spectrometry was performed at the Central Service for Experimental Research (SCSIE) of the University of Valencia. The proteomic data were analyzed in R with the Differential Enrichment analysis of the Proteomics data (DEP) package (Zhang et al., 2018).

## 3 Results

### 3.1 Reconstruction and main characteristics of the *Bartonella quintana*’s metabolic model

The first draft metabolic model of *B. quintana* str Toulouse was obtained using the SEED server of ModelSEED, based on a newly obtained annotation by RAST. The genome of *B. henselae* str. BM1374163 (Alsmark et al., 2004) was used to search for orthologous genes to fix some wrong annotations in genes involved in metabolic functions, thus helping to close some gaps using COBRApy (Ebrahim et al., 2013). This Python module enables the addition, deletion, and modification of each model element. These two organisms shared 95.3% of protein-coding sequences and 79.2% of collinear genes (Supplementary Figure S1). These similarities allowed for a more accurate and efficient search for the addition of metabolic reactions and the rapid reconstruction of the draft model for *B. quintana*, enabling us further to refine it into a well-curated, specific model.

Biomass composition can substantially affect fundamental model predictions (Beck et al., 2018), and for this reason, it needs to be integrated into metabolic models, which was also performed with COBRApy. The list of compounds included in the modified biomass equation, as well as their coefficients, are provided in Supplementary Table S1.

**FIGURE 2:**
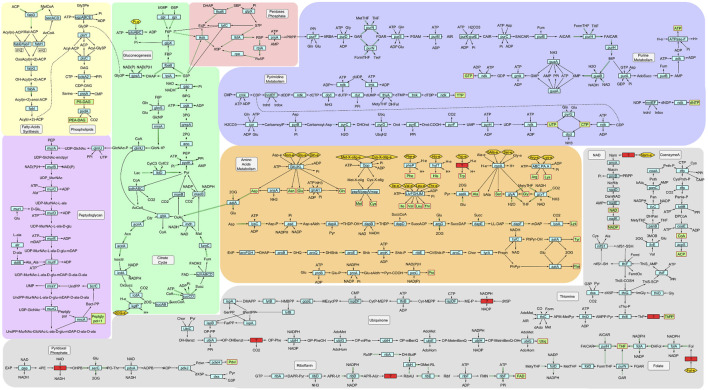
Metabolic chart of *B. quintana* str. Toulouse. Light-blue rectangular nodes: enzymes involved in each reaction; green rectangles with red border: essential compounds for growth; red rectangles: reactions needed with no gene for the corresponding enzyme identified in the genome; golden yellow hexagons: substrates that need to be imported. Green double-lined edges represent reversible reactions, showing only one of the reaction directions.

The genes and reactions included in the model were manually curated to get a refined metabolic model (Figure 2; Supplementary Table S2), composed of 300 protein-coding genes associated with metabolism (representing the 23.1% of the total of genes annotated in the genome) involved in 370 reactions, 49 of which are exchange reactions and 44 of which are involved in transport. 355 metabolites are included in the model, 343 of which (96.6%) are included in at least two reactions and 139 (30,1%) in three or more reactions. The quality analysis performed with MEMOTE assigned it a score of 85% (Supplementary Material S1), similar to the score obtained for other GEMs of model organisms (van ‘t Hof et al., 2022). The unrestricted model (Bquint GEM) predicts a growth rate of 17.1 1/h and the oxygen-restricted model (Bquint GEM O2) predicts a growth rate of 4.2 1/h.

Related to bioenergetics and carbon sources, we observed that there are no transporters for simple sugars like glucose or fructose. Furthermore, this organism has an incomplete glycolysis pathway, a fact that has also been observed in *B. henselae*, lacking both hexokinase (EC 2.7.1.1) and phosphofructokinase (EC 2.7.1.11) (Canback et al., 2002; Chenoweth et al., 2004). The remaining pathway reactions are still present in the network because they are reversible and also act in gluconeogenesis. This second pathway provides the precursors for the pentose phosphate pathway, which produces the ribulose phosphate needed for nucleotide synthesis. The Krebs cycle is complete, and it is the primary producer of reductive power (NADH).

The model includes 11 of the 20 proteogenic amino acids synthesis pathways (Ala, Asn, Asp, Gln, Glu, Gly, Lys, Phe, Pro, Ser, Tyr) and has specific transporters for another 6 (Arg, His, Ile, Leu, Thr, Val). There are no particular transporters for the remaining amino acids (Cys, Met, Trp), but they could be taken up through the ABC system for the import of dipeptides and oligopeptides. Subsequently, these compounds can be hydrolyzed to yield individual amino acids.

Regarding vitamins and cofactors, most of them have incomplete biosynthetic pathways. Pantothenate (vitamin B5), the precursor of coenzyme A, is the only one with a complete path, and only a few key enzymes are missing for the biosynthesis of NAD, pyridoxal phosphate, and ubiquinone. In the case of NAD, the salvage pathway is entirely functional, so this bacterium is capable of recycling intermediates for its synthesis. Any of these vitamins and cofactors have a specific transporter to incorporate them or any of the intermediates of the pathways.

In order to elucidate the essential compounds for the growth of *B. quintana* that could be incorporated from the culture medium, a comprehensive analysis of all reactions facilitating the transport of molecules from the extracellular environment was undertaken. In the absence of kinetics data, this analysis is sequence-based and aimed at improving the functional annotation of transporters encoded in the genome. Consequently, nine additional transport reactions that were absent from the original annotation were incorporated into the model. Among the added reactions, the branched-chain amino acid (BCAA) transporter and the heme group transporter stand out, as these compounds are pretty abundant in blood. The resulting model comprises 57 genes associated with transport that encode the proteins that constitute 25 transporters able to perform a total of 42 transport reactions. The proteins involved in the electron transport chain are not included. We have computed the flux variation in *in silico* mutants for each of these transporters, both individually and in combinations of two, to measure their impact on the growth of the organism. The outcomes have been categorized as essential, moderate, and non-essential based on the level of conservation in biomass production (<10%, 10%–80%, and >80%, respectively; Supplementary Table S3). We observed that 18 out of the 25 genes are essential, as their absence completely prevents bacterial growth, notably including transporters for most amino acids and ions. When a double mutant analysis was performed, there were no significant differences except for potassium transport. That is due to the presence of redundant transporters that share substrates and can complement each other. Thus, only in the absence of both does a significant deficit for the transported molecule occur in the cell, preventing its growth.

### 3.2 Composition of enriched media and validation

Based on the *in silico* knockout analyses, we searched for important compounds for cell growth that are present in low concentrations in commercial media and could be added to improve growth. We calculated the exchange fluxes in the model, which are linked to the transport reactions of molecules found in the medium, to incorporate them into the cell.

As a next step in the model reconstruction, we streamlined the overall flux of the models towards biomass production, obtaining a list of thirty-seven essential compounds from the environment required for the growth of the models (Table 1). Notably, in the absence of glucose or other basic sugar transporters, most influxes are associated with amino acids, as well as intermediates of the Krebs cycle. A high influx of 2-oxoglutarate and glutamine is observed, both of which are related to transamination reactions and nitrogen metabolism. It is noteworthy that glutamine and the BCAAs (valine, leucine, and isoleucine) have a relatively high concentration in the blood compared to other amino acids (Brody, 1999). The main differences we observed between Bquint GEM and Bquint GEM O2 are related to amino acid metabolism. When oxygen intake is restricted, aspartate, serine and D-alanine intake rises, while glutamate and glycine decreases. In the case of glutamate, the observed decrease is compensated because it participates in multiple transamination reactions, allowing its biosynthesis from aspartate and glutamine, which could explain the increase in aspartate intake. Regarding glycine, it plays a role in the one-carbon pool metabolism and it is synthesized from serine and folate derivatives. Lastly, D-alanine serves as a key precursor in bacterial cell wall biosynthesis and can be converted into L-alanine by alanine racemase (EC 5.1.1.1).

**TABLE 1:** Influxes and outfluxes of essential compounds in steady-state for *B. quintana* with no restrictions (Bquint GEM) and with controlled amounts of O_2_ (Bqint GEM O2).

Bquint GEM	
Metabolite	Flux (mmol/gDCW/h)
O_2_	351.2
H+	155.9
**2-Oxoglutarate**	**132.6**
L-Glutamate	19.67
L-Glutamine	17.71
Glycine	10.84
L-Serine	10.44
L-Leucine	7.488
L-Valine	7.046
L-Arginine	4.916
L-Isoleucine	4.829
L-Threonine	4.216
L-Asparagine	4.006
K+	3.176
L-Phenylalanine	3.079
Di-Methionine	2.581
L-Histidine	1.575
Di-Cysteine	1.539
L-Tryptophan	0.9447
Citrate	0.2541
Mg2+	0.1412
Fe3+	0.127
Fe2+	0.1093
Ca2+	0.0847
Cl-	0.0847
Na+	0.0847
Nicotinamide	0.03898
Folate	0.01526
**β-alanine**	**0.01314**
Cu2+	0.01153
Mn2+	0.01125
Zn2+	0.005542
**Thiamin**	**0.003814**
Hemin	0.003814
Biotin	3.42E-05
H_2_O	−266.3
CO_2_	−526.2

Bquint GEM O2	
Metabolite	Flux (mmol/gDCW/h)
H+	36.9
O_2_	15
**2-Oxoglutarate**	**13.79**
L-Aspartate	6.495
L-Serine	5.218
L-Glutamine	4.344
L-Leucine	1.836
L-Valine	1.728
D-Alanine	1.672
L-Arginine	1.206
L-Isoleucine	1.184
L-Threonine	1.034
L-Asparagine	0.9826
K+	0.7789
L-Phenylalanine	0.7551
Di-Methionine	0.633
L-Histidine	0.3862
Di-Cysteine	0.3774
L-Tryptophan	0.2317
Citrate	0.06232
Mg2+	0.03462
Fe3+	0.03116
Fe2+	0.0268
Ca2+	0.02077
Cl-	0.02077
Na+	0.02077
Nicotinamide	0.00956
Folate	0.003742
**β-alanine**	**0.003222**
Cu2+	0.002827
Mn2+	0.00276
Zn2+	0.001359
**Thiamin**	**0.0009354**
Hemin	0.0009354
Biotin	8.39E-06
H_2_O	−44.77
CO_2_	−44.96

Positive values indicate compounds that the cell takes from the environment to grow; negative values indicate exported compounds. The four components that are defective in the commercial growth medium for *B. quintana* appear in bold.

When comparing these 37 essential compounds with the composition of Columbia agar base and the blood used in the standard preparation of culture media for blood-feeding microorganisms, we observed that there are four compounds absent in this medium: 2-oxoglutarate, thiamine, folate, and β-alanine. In the case of 2-oxoglutarate, its input flux is the second highest after oxygen, which reflects its relevance in the growth of this organism. Additionally, this compound is a crucial element in sugar and amino acid metabolism. The other three compounds have much smaller fluxes but participate in very important reactions in bacterial physiology.

We selected three out of these four compounds for the experimental validation of an improved growth media for *B. quintana*, to simplify the validation protocol. β-alanine was excluded because preliminary tests showed no changes in *Bartonella*’s growth performance when it was added individually (data not shown).

Seven different types of supplemented chocolate agar plates were produced, by adding the three selected compounds separately (2-oxogulutarate, K; folate, F; thiamine, T), in paired combinations (FK, FT, and KT), altogether (FKT), and the protocol described in Figure 1 was followed to check for growth differences between them. OD_600_ measurements were performed after 7 days of incubation. The experiment was repeated six times. Growth problems were observed in the plates inoculated from the 10^–4^ dilution of the original culture, with instances where none of the replicates in an experimental condition showed bacterial growth. That led us to exclude these data from the statistical analysis due to the lack of sufficient measurements.

**FIGURE 3:**
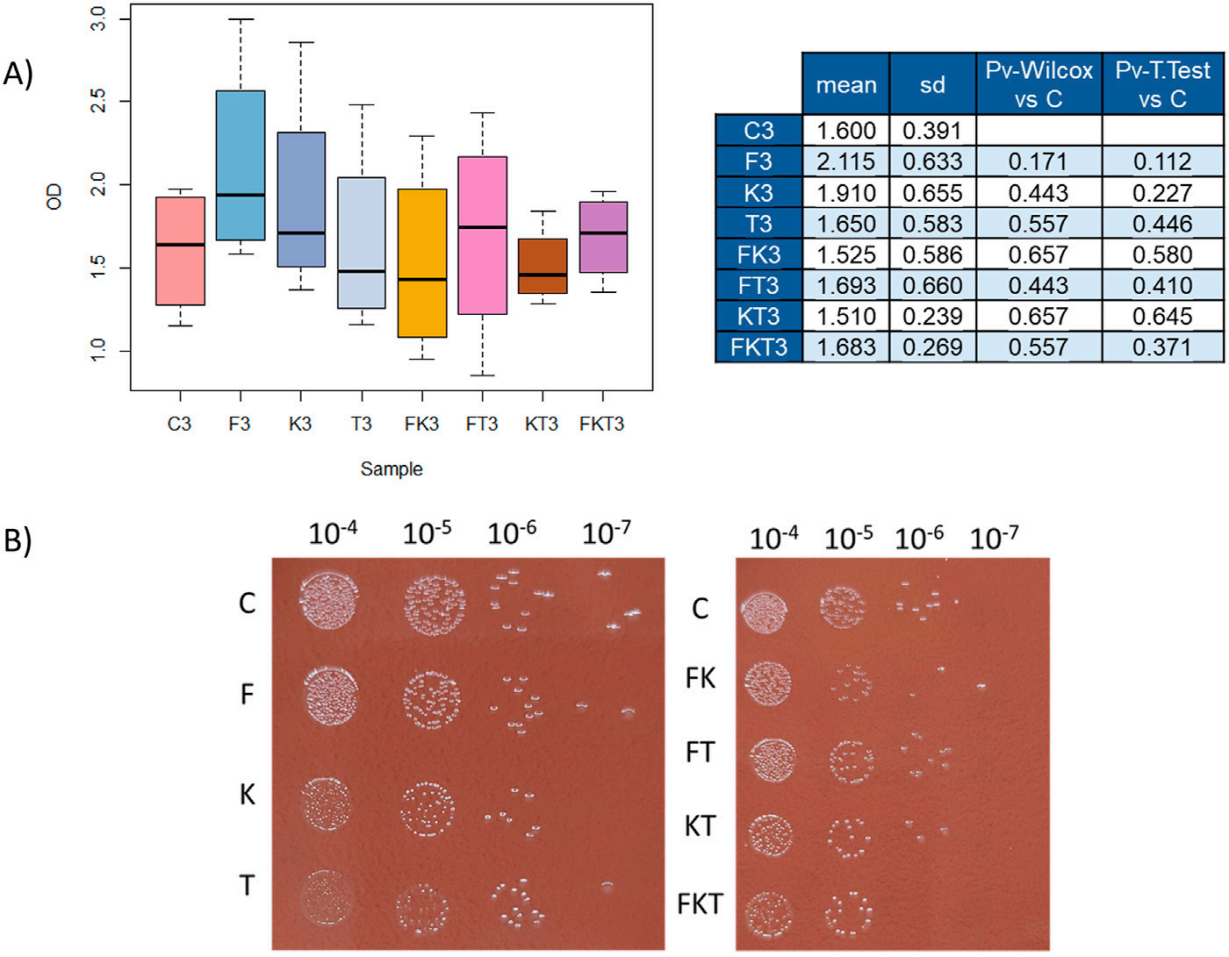
Growth and viability comparison analysis. **(A)** Boxplot of OD_600_ measurements from the different supplemented media after 7 days of growth on plate of the dilution 10^–3^ (indicated with a number −3 in Figure 1). Statistical analysis against control condition is represented in the adjacent table, with each corresponding p-value. **(B)** Viability comparison between supplemented media. C, control; F, folate; K, 2-oxoglutarate; T, thiamine.

When comparing the data from plates inoculated from the 10^–3^ dilution to control chocolate agar plates, no significant differences were observed in any of the seven types of plates (Figure 3A). During the analysis of these data, some replicates had to be discarded because the initial cultures used for comparison had an OD_600_ above 2. In these cases, the dilutions reached saturation and generated inconsistencies between technical replicates from each medium. All data, including that from the discarded experiments, are provided in Supplementary Table S4.

Cell viability was also measured to compare the outcome in chocolate agar and supplemented media. A cell count was performed after 7 days to do so, starting with an adjusted OD_600_ for all media (Figure 3B). Surprisingly, the viability in media with a single added compound (F, K, T) barely differs from the viability in control media. It is similar or slightly lower in the media with two compounds added (FK, FT, and KT), and, in the case of the medium with all three compounds added (FKT), there is a decrease of nearly two orders of magnitude in the number of cells.

### 3.3 Proteomic analysis

**FIGURE 4:**
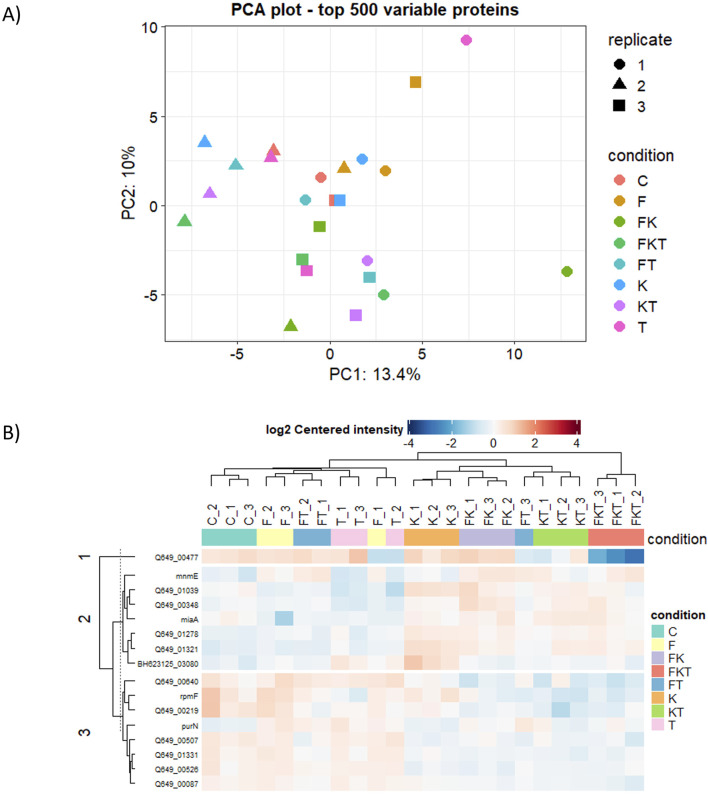
Comparative proteomics analysis of *B. quintana* in different growth conditions. **(A)** Principal components analysis of the top 500 proteins. **(B)** Clustering analysis of significantly differentially-expressed proteins.

We also analyzed if there were differences in protein levels among *B. quintana* cultures grown in the different media used in this study. We identified 820 proteins by mass spectrometry. A principal component analysis (PCA) of the top 500 proteins (Figure 4A) from our samples revealed differences among replicates of the same condition, which correlated with our previous growth experiments, where the variance among biological replicates was high, making it challenging to observe significant differences between the samples. Nevertheless, 16 of the identified proteins appeared to be differentially expressed compared to the control (Table 2). Proteins with significant changes are defined by user-defined cut-offs based on the adjusted p-value, which should be lower than 0.05, and the absolute fold-change, which should be larger than 0.5. The cut-off of 0.5 is roughly corresponding to the standard deviation of the fold changes in the FKT vs. C comparison.

**TABLE 2:** List of over and under-expressed proteins in *B. quintana* in each media compared with the control medium.

Name	ID	Description	K vs. C	FK vs. C	KT vs. C	F vs. C	FT vs. C	T vs. C	FKT vs. C
BH623125_03080	A0A5S9EVU9_BARQI	type III PLP-dependent enzyme/ornithine decarboxylase	1.14					0.682	
miaA	MIAA_BARQU	tRNA dimethylallyltransferase				−0.727			
mnmE	MNME_BARQU	tRNA modification GTPase MnmE		0.867		0.609	0.848		0.72
purN	A0A0H3M0N7_BARQU	Phosphoribosylglycinamide formyltransferase				0.631	0.628		
Q649_00087	A0A0H3M3W4_BARQU	Cytoplasmic protein							−0.525
Q649_00219	A0A5S9EW29_BARQI	Lipoprotein		−0.894	−1.36		−0.78		−0.951
Q649_00348	A0A0H3LT42_BARQU	Heat shock protein ibpA1		0.626					
Q649_00477	A0A0H3LTD2_BARQU	NlpC/P60 domain-containing protein							−3.04
Q649_00507	A0A0H3LTE9_BARQU	phospho-N-acetylmuramoyl-pentapeptide-transferase	−0.619				−0.555		−0.681
Q649_00526	A0A0H3M285_BARQU	Cell wall hydrolase SleB domain-containing protein		−0.535					
Q649_00640	A0A0H3M033_BARQU	Lectin-like protein BA14k							−0.943
Q649_01039	A0A0H3M2P0_BARQU	Small heat shock protein ibpA2						−0.83	
Q649_01278	A0A0H3LWB3_BARQU	N-acetylmuramoyl-L-alanine amidase	0.693						
Q649_01321	A0A0H3LWF2_BARQU	Calcineurin-like phosphoesterase domain-containing protein	1.05	0.694	0.633				0.662
Q649_01331	A0A0H3LWF7_BARQU	Beta-lactamase hydrolase-like protein phosphatase-like domain-containing			−0.57				
rpmF	RL32_BARQU	Large ribosomal subunit protein bL32			−1.22				−1.06

All fold change values are in log_2_. Green and orange cells represent over- and under-expressed proteins, respectively, setting a fold change threshold based on the standard deviation of samples.

A clustering analysis of these differentially expressed proteins (Figure 4B) revealed that all media containing 2-oxoglutarate grouped (cluster K). This group includes proteins related to cell wall modification and tRNA modifications, as well as changes in proteins involved in the cell cycle, cell adhesion, and morphology. There are also some stress-related proteins and antioxidant synthesis. There are not many differences between the control (C) and those media supplemented with folate (F) and thiamine (T). A slight increase in proteins involved in purine biosynthesis is observed, likely related to ATP and GTP synthesis (PurN), as well as proteins associated with RNA metabolism (MiaA, MnmE). Proteins related to cell wall formation (Q649_00507, Q649_00526, Q649_00219) are underexpressed. In contrast, those related to cell wall synthesis and turnover (Q649_01321, Q649_01278) are overexpressed in cluster K. Triple compound medium (FKT) only differs from cluster K due to a highly underexpressed protein, an NPLC/P60 endopeptidase related to cell growth and morphology that causes cell aggregation (Duchêne et al., 2019).

## 4 Discussion

The biomass composition represents the essential components needed for the growth of the organism of interest. This composition is usually integrated into metabolic models using a biomass reaction. This theoretical reaction summarizes all the reactants and products that the cell needs to be able to grow normally. The selection of a biomass reaction that aligns with the nutritional needs of the organism under study is crucial. However, to study non-model organisms such as *Bartonella,* which lack specific information or information from closely related species or genera, researchers often define generalist biomass reactions that tend to be very permissive in terms of biomass production. When comparing the general reaction provided by ModelSEED with the biomass reaction from the iJO1366 model of *E. coli*, significant changes were observed in the fluxes of reactions involved in biomass production. Given that *B. quintana* is a fastidious organism with complex nutritional requirements, our model was based on a more restrictive biomass reaction than that of the iJO1366 model in terms of the stoichiometry and components involved.

A key limitation of the reconstructed model is that its quantitative predictions lack physiological accuracy, as reflected in the unrealistically high flux values obtained in some simulations. In particular, the model predicts a maximum growth rate of 4.2 h^-1^, corresponding to a doubling time of approximately 10 min. This value is highly improbable when compared to some of the fastest-growing bacteria known. For instance, *V. natriegens* exhibits a maximum reported growth rate of 1.7 h^-1^ (doubling time of ∼20 min) under optimal laboratory conditions (Long et al., 2017). Even the thermophilic extremophile *Geobacillus LC300*, which thrives at high temperatures and has been described as one of the fastest-growing aerobic bacteria, reaches a maximum growth rate of only 2.15 h^-1^ in glucose-based media (Cordova et al., 2015). Given that the organism modeled in this study grows significantly more slowly than both *V. natriegens* and *G. LC300*, this overestimation suggests that some constraints in the model are insufficient to accurately reflect the organism’s physiological limitations.

This discrepancy likely arises from the lack of experimentally determined kinetic parameters for key transporters and enzymatic reactions, which limits the model’s ability to constrain metabolic fluxes realistically. The experimental acquisition of such kinetic data is particularly challenging in this case, as the organism grows at a very slow rate and requires Biosafety Level 2 (BSL-2) containment, significantly complicating *in vivo* flux measurements and enzyme kinetics characterization. Consequently, the model’s quantitative predictions should be interpreted with caution.

Regarding the primary carbon source in the model, we observed that there are no transporters associated with the basic sugars typically used by bacteria, such as glucose or fructose. Both *B. quintana* and *B. henselae* have the *ptsH* and *fruB* genes, encoding the general (non-sugar specific) phosphocarrier protein HPr and the multiphosphoryl transfer protein of the phosphotransferase system (PTS) fructose-mannitol family, respectively. Additionally, the gene *manX* coding for the IIAB subunit of the mannose-specific PTS transporter is present, which has been shown to have an affinity for other sugars, albeit with a lower transport rate (Rephaeli and Saier, 1980). However, for this transporter to be functional, the other two permease subunits, IIC and IID, need to be present, and there is no evidence of these genes or any homologous ones in the genome of *B. quintana* Toulouse, which suggests that other compounds may serve as a carbon source in this metabolic model. One possibility is that *B. quintana* uses amino acids as a source of both carbon and nitrogen, a phenomenon observed in various bacteria (Jorgensen et al., 1993; Li et al., 2011; Babaei et al., 2014). Among the proteinogenic amino acids, glutamate stands out as it actively participates in many transamination reactions and is an important element in nitrogen metabolism (Walker and van der Donk, 2016). Glutamate, when used as a single carbon source, has been shown to support growth in a diverse number of organisms at a rate comparable to sugars from conventional media (Halvorson, 1972). In our model, *in silico* simulations of transporter knockouts indicated that glutamate consumption by the cell increased significantly when other transporters were eliminated, particularly in the knockouts of the 2-oxoglutarate transporter. It is worth noting that 2-oxoglutarate is the second candidate as a carbon source in this organism, since it showed a high input flux in the metabolic model, but its presence was not mentioned in the culture medium composition. It is a versatile compound within bacterial metabolism, featuring various pivotal pathways within the organism (Huergo and Dixon, 2015). Among its roles, 2-oxoglutarate serves as a crucial intermediate in the Krebs cycle, holds significance in anaplerotic pathways, and stands out as one of the most relevant compounds in nitrogen metabolism due to its involvement in the GS-GOGAT cycle.

When we studied the model fluxes associated with this compound, a notably high flux in the Krebs cycle was observed, generating a large amount of reducing power that feeds into ATP production via the electron transport chain, as expected. The two key compounds in our model associated with this pathway are 2-oxoglutarate and oxaloacetate. The latter compound branches into two main pathways: the first continues the Krebs cycle, converting into citrate through citrate synthase (EC 2.3.3.1), and the second generates pyruvate via malic enzyme (EC 1.1.1.40). In this way, it supplements the gluconeogenesis pathway, leading to the formation of glyceraldehyde-3-phosphate (G3P). G3P further branches into the synthesis of glycerol-3-phosphate (Gly3P) for phospholipid production and fuses with dihydroxyacetone phosphate (DHAP) to form fructose-6P (F6P), which then feeds into the pentose phosphate pathway to form the nucleotide backbone. However, this process creates a deficit of Krebs cycle intermediates, which requires compensation by incorporating 2-oxoglutarate from the medium, explaining the high intake flux needed for the model. Additionally, oxaloacetate undergoes transamination from glutamate via the enzyme aspartate aminotransferase (EC 2.6.1.1), generating 2-oxoglutarate and aspartate. That is in concordance with the glutamate transporter flux mentioned above.

As we could see in the proteomic analysis, those media supplemented with 2-oxoglutarate had a different expression pattern than the other tested media, mostly affecting membrane and cell wall proteins. We observed a depletion in enzymes related to peptidoglycan synthesis, like phospho-N-acetylmuramoyl-pentapeptide-transferase (EC 2.7.8.13). Still, there are other proteins related to cell wall synthesis and modification that are overexpressed. There are proteins related to the cell cycle, like heat shock protein ibpA1, associated with lipoproteins involved in cell division (Tao et al., 2015). These results could indicate that the addition of 2-oxoglutarate to the medium induces significant changes in the cell envelope that produce alterations in the cell cycle, which could affect growth and viability.

Additionally, it was observed that when the three compounds (FKT) were added, the pattern was very similar with a single difference: the downregulation of the NPLC/P60 endopeptidase protein. The absence of this protein, which is associated with the cell cycle and membrane modification, results in rounded cell shapes and increased cell aggregation (Duchêne et al., 2019), as well as the appearance of multiple septa during the cytokinesis process (Squeglia et al., 2019). These changes in the membrane may be affecting the viability in this culture medium, as shown in Figure 3B.

Even though there is no evidence of the presence of genes encoding transporters for thiamine and folate in the genome of *B. quintana*, our model includes these transport reactions because these two vitamins are needed to synthesize cofactors essential for growth. Thiamine is a precursor of thiamine pyrophosphate (ThPP), which acts as a cofactor in important reactions of the glycolysis and the Krebs cycle, such as those catalyzed by 2-oxoglutarate dehydrogenase and pyruvate dehydrogenase (Tittmann, 2009). Typically, cells take up unphosphorylated thiamine (Jaehme and Slotboom, 2015) and have mechanisms for synthesizing ThPP directly from it using a thiamine pyrophosphokinase (EC: 2.7.6.2) (Nosakat et al., 1993; Fankhauser et al., 1995). *B. quintana*, possesses the gene encoding this enzyme, but there is no evidence of this kind of transporter. Other organisms are able to synthesize ThPP using thiamine monophosphate (ThMP) as an intermediate (Rodionov et al., 2002). In *B. quintana* this biosynthetic pathway is nearly complete, up to the production of ThMP, but there is no evidence of the presence of a kinase able to produce ThPP from it.

Folate is a precursor of tetrahydrofolate (THF), a compound that participates in several carbon group transfer reactions in the biosynthesis of purines, pyrimidines, and methionine (Shetty and Varshney, 2021). The biosynthetic pathway of folate derivatives is complete. Still, no transporter allowing the entry of this molecule or any of its derivatives into the cell has been identified in bacteria from the genus *Bartonella*. The proteomic data related to media with folate in them suggest an increase in nucleotide and RNA metabolism activity. which supports the idea that folate is being incorporated into the cell, even though there is no evidence of a folate transporter.

The differences observed among the supplemented and control media are not significant, probably due to the high variability observed in the measurements of each replicate. One of the main problems that can lead to this considerable variation is the autoaggregation experienced by *B. quintana* when suspended in liquid media (Zhang et al., 2004), which results in non-uniform cell distribution during the steps of cell resuspension, leading to a bias in cell concentration in the different dilutions.

Additional problems also arise with high cell concentrations. When the initial concentration is high, we observed that bacteria exhibit overall lower growth and viability. Although no experimental evidence has been described in *Bartonella*, in other prokaryotic organisms, this phenomenon is attributed to contact inhibition related to the presence of type 5 and type 6 secretion systems (T5SS and T6SS, respectively) (Ikryannikova et al., 2020). *B. quintana* presents a type 4 secretion system (T4SS). This system can penetrate the cytoplasm of other Gram-negative bacteria and can be used to secrete toxic compounds to eliminate competing bacteria (Sgro et al., 2019). This T4SS has a similar structure to the molecular syringe of T6SS (Green and Mecsas, 2016). All these characteristics could cause *B. quintana* to exhibit lower growth at high bacterial concentrations.

In conclusion, the reconstruction of the metabolic model of *B. quintana* identified critical pathways and essential compounds for its growth in culture, highlighting 2-oxoglutarate as a key source of carbon and energy. This model represents a significant step towards optimizing *B. quintana* as a chassis for synthetic biology applications, although a deeper understanding of its metabolism is needed. The model remains a useful qualitative tool for investigating the organism’s metabolic capabilities. While absolute flux values may not be reliable, the model provides a framework for identifying gaps in the current understanding of its metabolism. By contextualizing these limitations, the model can serve as a valuable starting point for guiding future experimental studies. The development of an optimal culture medium that facilitates laboratory work and precedes its possible use as a chassis in synthetic biology has been hampered by the absence of a defined carbon source optimal for the organism and the lack of knowledge of the vitamin transporters predicted by the model, which raises the need to carry out future *in vitro* studies that would allow the model to be improved.

## Data Availability

The datasets presented in this study can be found in online repositories. The names of the repository/repositories and accession number(s) can be found below: https://github.com/Emigarsan/Bartonella_analysis.git.

## Supplementary material

SUPPLEMENTARY FIGURE S1 Comparison of *B. quintana* and *B. henselae* genomes. **(A)** Alignment of genomic sequences. **(B)** Venn diagram of the cluster of protein-coding sequences.

SUPPLEMENTARY TABLE S1 Biomass equation components, with their stoichiometric coefficients. Negative values represent the reactants and positive values represent products. Bquint_biomass_coefficients.xlsx.

SUPPLEMENTARY TABLE S2 Reactions present in the metabolic network of *B. quintana*. Reactions added to the metabolic model with an identified gene are highlighted in blue and has the id tag “rxna”. Those reactions without a gene associated are highlighted in green and has the id tag “rxnp”. The flux values refer to the model with controlled amounts of O_2_ (Bqint GEM O2). Bquint_fluxes.xlsx file.

SUPPLEMENTARY TABLE S3 Ratio of conservation in biomass production for transport *in silico* mutants from the model. Table_Transport_Mutants.xlsx file.

SUPPLEMENTARY TABLE S4 Average OD600 measurements from supplemented media comparison experiment. These data are retrieved from plates inoculated from the 10^–3^ dilution of original culture. OpticalDensity_media.xlsx file.

SUPPLEMENTARY MATERIAL S1 MEMOTE complete report from *B. quintana*’s GEM. Bquint_Report_MEMOTE.pdf.
