# Supplementary figures and images for: Determination of the genome-scale metabolic network of Bartonella quintana str. Toulouse to optimize growth for its use as chassis for synthetic biology
Source: Front Bioeng Biotechnol. 2025 Mar 27;13:1527084. doi: 10.3389/fbioe.2025.1527084 (PMC11983613; doi:10.3389/fbioe.2025.1527084)

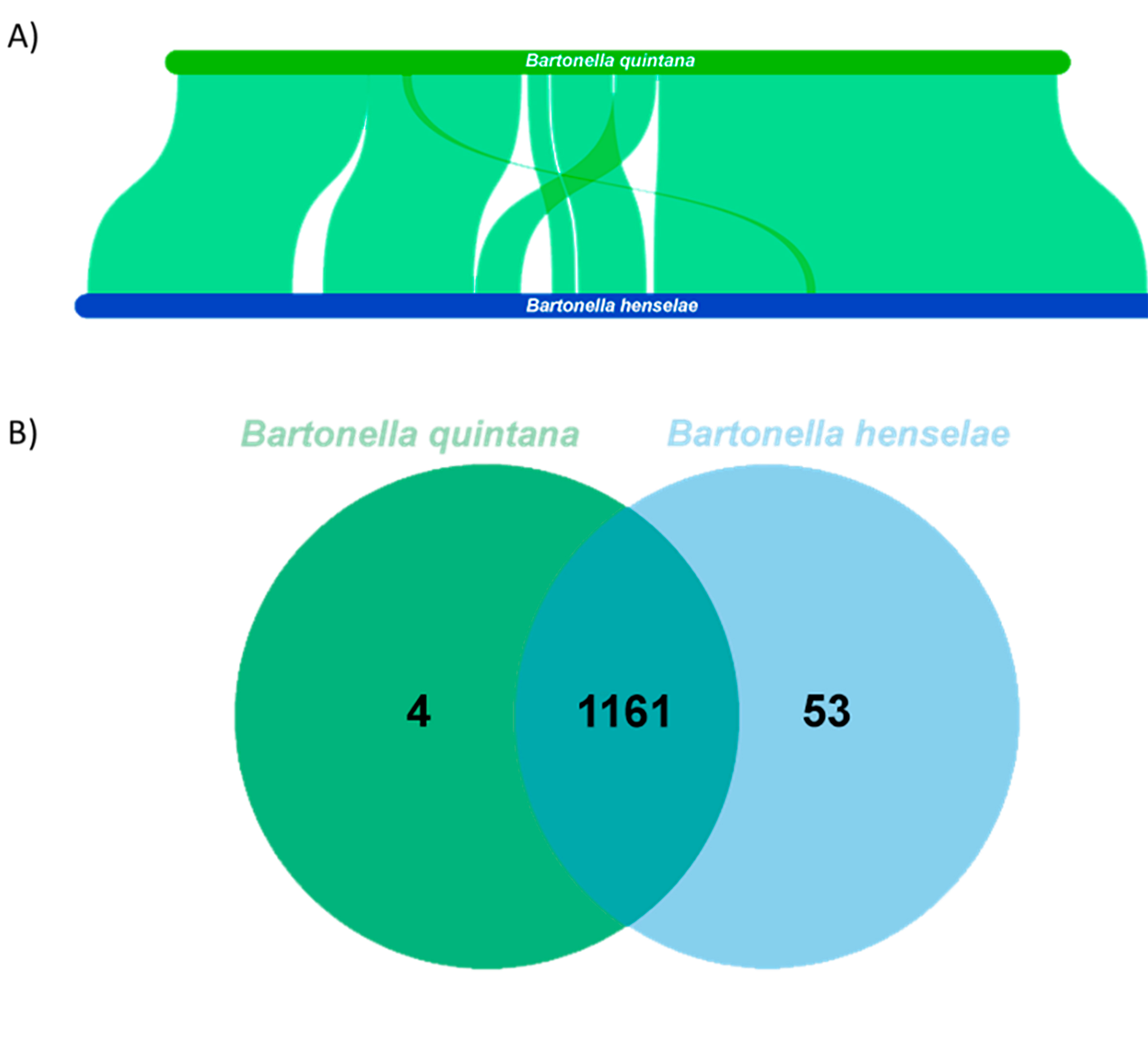

Supplement: Supplementary file 3 [file Image1.jpeg]
