# Supplementary material for: Determination of the genome-scale metabolic network of Bartonella quintana str. Toulouse to optimize growth for its use as chassis for synthetic biology
Source: Front Bioeng Biotechnol. 2025 Mar 27;13:1527084. doi: 10.3389/fbioe.2025.1527084 (PMC11983613; doi:10.3389/fbioe.2025.1527084)

Independent Section

Contains tests that are independent of the class of modeled organism, a model's complexity or types of identifiers that are used to describe its components. Parameterization or initialization of the network is not

Consistency

Stoichiometric Consistency	100.0%	x3
Mass Balance	74.5%	
Charge Balance	92.1%	
Metabolite Connectivity	100.0%	
Unbounded Flux In Default Medium	99.1%	
Sub Total	95%	x3

Annotation - Metabolites

Presence of Metabolite Annotation	100.0%
Metabolite Annotations Per Database	Info
pubchem.compound	68.5%
kegg.compound	81.6%
seed.compound	0.0%
inchikey	72.7%
inchi	72.7%
chebi	64.9%
hmdb	0.0%

Specific Section

Covers general statistics and specific aspects of a metabolic network that are not universally applicable. See readme for more details.

SBML

SBML Level and Version	SBML Level 3 Version 1
FBC enabled	true

Basic Information

Model Identifier	A_283165_10
Total Metabolites	359
Total Reactions	370
Total Genes	300
Total Compartments	2
Metabolic Coverage	1.23
Unconserved Metabolites	0
Minimal Inconsistent Net Stoichiometries	0

Metabolite Information

Unique Metabolites	315
Duplicate Metabolites in Identical Compartments	0

reactome	0.0%
metanetx.chemical	85.2%
bigg.metabolite	82.7%
biocyc	78.6%
Metabolite Annotation Conformity Per Database	Info
pubchem.compound	100.0%
kegg.compound	97.6%
seed.compound	0.0%
inchikey	100.0%
inchi	100.0%
chebi	100.0%
hmdb	0.0%
reactome	0.0%
metanetx.chemical	99.7%
bigg.metabolite	100.0%
biocyc	98.6%
Uniform Metabolite Identifier Namespace	100.0%
Sub Total	82%

Annotation - Reactions

Presence of Reaction Annotation	100.0%
Reaction Annotations Per Database	Info
rhea	56.5%

Metabolites without Charge	0
Metabolites without Formula	53
Medium Components	47

Reaction Information

Purely Metabolic Reactions	274
Purely Metabolic Reactions with Constraints	19
Transport Reactions	44
Transport Reactions with Constraints	1
Reactions With Partially Identical Annotations	0.03
Duplicate Reactions	0.00
Reactions With Identical Genes	0.41

Gene-Protein-Reaction (GPR) Associations

Reactions without GPR	1
Fraction of Transport Reactions without GPR	0.00
Enzyme Complexes	Errored

Biomass

Biomass Reactions Identified	1
Biomass Consistency	Errored
Biomass Production In Default Medium	3.99
Unrealistic Growth Rate In Default Medium	true

kegg.reaction	63.2%	
seed.reaction	0.0%	
metanetx.reaction	77.0%	
bigg.reaction	67.6%	
reactome	0.0%	
ec-code	0.0%	
brenda	0.0%	
biocyc	60.0%	
Reaction Annotation Conformity Per Database	Info	
rhea	100.0%	
kegg.reaction	100.0%	
seed.reaction	0.0%	
metanetx.reaction	100.0%	
bigg.reaction	99.5%	
reactome	0.0%	
ec-code	0.0%	
brenda	0.0%	
biocyc	100.0%	
Uniform Reaction Identifier Namespace	100.0%	
Sub Total	73%	

Annotation - Genes

Presence of Gene Annotation	100.0%

Biomass Production In Complete Medium	3.99
Blocked Biomass Precursors In Default Medium	2
Blocked Biomass Precursors In Complete Medium	2
Ratio of Direct Metabolites in Biomass Reaction	0.32
Number of Missing Essential Biomass Precursors	1

Energy Metabolism

Non-Growth Associated Maintenance Reaction	0
Growth-associated Maintenance in Biomass Reaction	true
Number of Reversible Oxygen-Containing Reactions	1
Erroneous Energy-generating Cycles	Info
MNXM3	331
MNXM63	8
MNXM51	4
MNXM121	8
MNXM423	Skipped
MNXM6	0
MNXM10	0
MNXM38	0
MNXM208	Skipped
MNXM191	0
MNXM223	Skipped
MNXM7517	Skipped

Gene Annotations Per Database	Info
refseq	91.0%
uniprot	88.7%
ecogene	0.0%
kegg.genes	91.0%
ncbigi	0.0%
ncbigene	10.7%
ncbiprotein	91.0%
ccds	0.0%
hprd	0.0%
asap	0.0%

Gene Annotation Conformity Per Database	Info
refseq	100.0%
uniprot	98.7%
ecogene	0.0%

There are no gene annotations for the ecogene database.

```
[]
```

kegg.genes	100.0%
ncbigi	0.0%
ncbigene	100.0%
ncbiprotein	100.0%
ccds	0.0%

MNXM12233	Skipped
MNXM558	Skipped
MNXM21	10
MNXM89557	0

Network Topology

Universally Blocked Reactions	13
Orphan Metabolites	3
Dead-end Metabolites	1
Stoichiometrically Balanced Cycles	5
Metabolite Production In Complete Medium	55
Metabolite Consumption In Complete Medium	62

Matrix Conditioning

Ratio Min/Max Non-Zero Coefficients	0.00
Independent Conservation Relations	10
Rank	349
Degrees Of Freedom	21

Experimental Data Comparison

Growth Prediction	Skipped
Gene Essentiality Prediction	Skipped

Misc. Tests

hprd	0.0%	
asap	0.0%	

Sub Total	62%

Annotation - SBO Terms

Metabolite General SBO Presence	100.0%
Metabolite SBO:0000247 Presence	100.0%
Reaction General SBO Presence	100.0%
Metabolic Reaction SBO:0000176 Presence	100.0%
Transport Reaction SBO:0000185 Presence	100.0%
Exchange Reaction SBO:0000627 Presence	100.0%
Demand Reaction SBO:0000628 Presence	Skipped
Sink Reactions SBO:0000632 Presence	Skipped
Gene General SBO Presence	100.0%
Gene SBO:0000243 Presence	100.0%
Biomass Reactions SBO:0000629 Presence	100.0%

Sub Total	82%

Total Score	85%

Total Score

Environment

Python Version	3.12.7
Platform	Linux
Memote Version	

85%

Score per Category

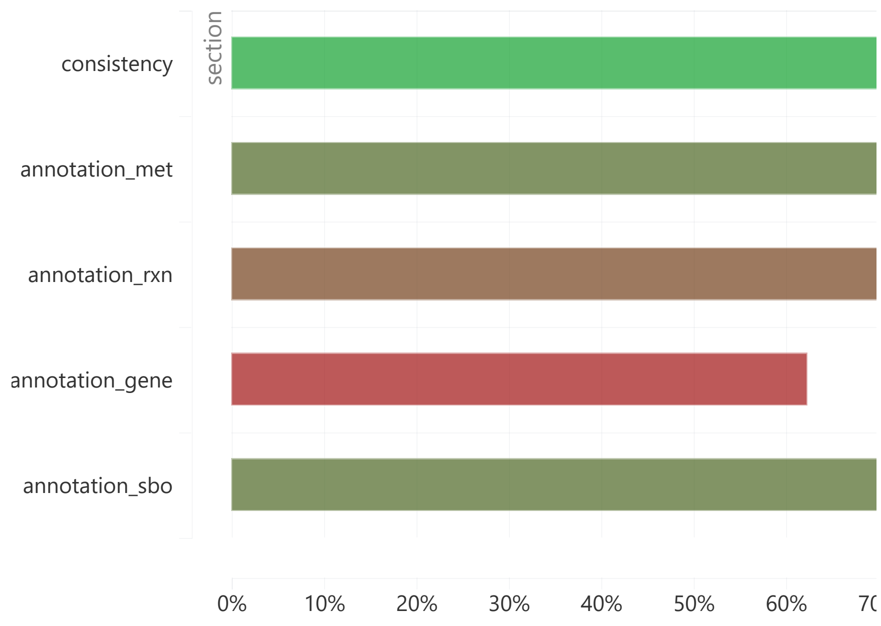

Supplement: Supplementary file 4 [file DataSheet1.pdf]
